# Thallium(I) copper(I) thorium(IV) tris­elenide, TlCuThSe_3_

**DOI:** 10.1107/S1600536812026669

**Published:** 2012-06-20

**Authors:** Lukasz A. Koscielski, James A. Ibers

**Affiliations:** a Department of Chemistry, Northwestern University, 2145 Sheridan Road, Evanston, IL 60208-3113, USA

## Abstract

Thallium(I) copper(I) thorium(IV) tris­elenide, TlCuThSe_3_, crystallizes with four formula units in the space group *Cmcm* in the KCuZrS_3_ structure type. There is one crystallographic­ally independent Th, Tl, and Cu atom at a site of symmetry 2/*m*.., *m*2*m*, and *m*2*m*, respectively. There are two crystallographically independent Se atoms at sites of symmetry *m*.. and *m*2*m*. The structure consists of sheets of edge-sharing ThSe_6_ octa­hedra and CuSe_4_ tetra­hedra stacked parallel to the (010) face, separated by layers filled with chains of Tl running parallel to [100]. Each Tl is coordinated by a trigonal prism of Se atoms.

## Related literature

For compounds of type *AMM*’*Q*_3_, see: Pell & Ibers (1996 ▶); Klepp & Gurtner (1996 ▶) for *A* = Tl; Pell *et al.* (1997 ▶); Yao *et al.* (2008 ▶); Wells *et al.* (2009 ▶) for *M* = Ag; Bugaris & Ibers (2009 ▶) for *M* = Au; Mansuetto *et al.* (1993 ▶); Pell & Ibers (1996 ▶) for *M*′ = Ti; Mansuetto *et al.* (1992 ▶, 1993 ▶); Huang *et al.* (2001 ▶); Pell *et al.* (1997 ▶) for *M*′ = Zr; Klepp & Sturmayr (1997 ▶, 1998 ▶); Pell *et al.* (1997 ▶) for *M*′ = Hf; Seldy *et al.* (2005 ▶); Narducci & Ibers (2000 ▶) for *M*′ = Th; Yao *et al.* (2008 ▶); Sutorik *et al.* (1996 ▶); Bugaris & Ibers (2009 ▶); Huang *et al.* (2001 ▶); Cody & Ibers (1995 ▶) for *M*′ = U; Wells *et al.* (2009 ▶) for *M*′ = Np. For computational details, see: Gelato & Parthé (1987 ▶). For additional synthetic details, see: Witt *et al.* (1956 ▶).

## Experimental

### 

#### Crystal data

TlCuThSe_3_
*M* *_r_* = 736.83
Orthorhombic, 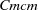

*a* = 4.1678 (2) Å
*b* = 14.2227 (7) Å
*c* = 10.8476 (5) Å
*V* = 643.02 (5) Å^3^
*Z* = 4
Mo *K*α radiation
μ = 68.19 mm^−1^
*T* = 100 K
0.10 × 0.07 × 0.02 mm

#### Data collection

Bruker APEXII CCD diffractometer
Absorption correction: numerical (*SADABS*; Sheldrick, 2008*b* ▶) *T*_min_ = 0.101, *T*_max_ = 0.489
7476 measured reflections
474 independent reflections
451 reflections with *I* > 2σ(*I*)
*R*_int_ = 0.032

#### Refinement

*R*[*F* ^2^ > 2σ(*F* ^2^)] = 0.021
*wR*(*F* ^2^) = 0.046
*S* = 1.59
474 reflections
24 parameters
Δρ_max_ = 2.01 e Å^−3^
Δρ_min_ = −1.34 e Å^−3^

### 

Data collection: *APEX2* (Bruker, 2009 ▶); cell refinement: *SAINT* (Bruker, 2009 ▶); data reduction: *SAINT*; program(s) used to solve structure: *SHELXS97* (Sheldrick, 2008*a* ▶); program(s) used to refine structure: *SHELXL97* (Sheldrick, 2008*a* ▶); molecular graphics: *CrystalMaker* (Palmer, 2009 ▶); software used to prepare material for publication: *SHELXL97*.

## Supplementary Material

Crystal structure: contains datablock(s) I, global. DOI: 10.1107/S1600536812026669/wm2644sup1.cif

Structure factors: contains datablock(s) I. DOI: 10.1107/S1600536812026669/wm2644Isup2.hkl

Additional supplementary materials:  crystallographic information; 3D view; checkCIF report

## Figures and Tables

**Table 1:** Selected bond lengths (Å)

Th1—Se2^i^	2.8844 (4)
Th1—Se2	2.8844 (4)
Th1—Se1^ii^	2.9057 (5)
Th1—Se1^iii^	2.9057 (5)
Th1—Se1^iv^	2.9057 (5)
Th1—Se1^v^	2.9057 (5)
Tl1—Se2^vi^	3.2831 (9)
Tl1—Se2^vii^	3.2831 (9)
Tl1—Se1^viii^	3.3564 (6)
Tl1—Se1^vi^	3.3564 (6)
Tl1—Se1^ix^	3.3564 (6)
Tl1—Se1^vii^	3.3564 (6)
Cu1—Se1	2.4617 (11)
Cu1—Se1^x^	2.4617 (11)
Cu1—Se2^vii^	2.5517 (11)
Cu1—Se2^vi^	2.5517 (11)

Symmetry codes: (i) 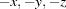; (ii) 
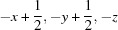
; (iii) 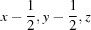; (iv) 
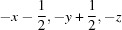
; (v) 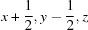; (vi) 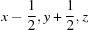; (vii) 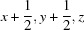; (viii) 
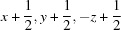
; (ix) 
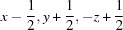
; (x) 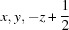.

**Fig. 1.**
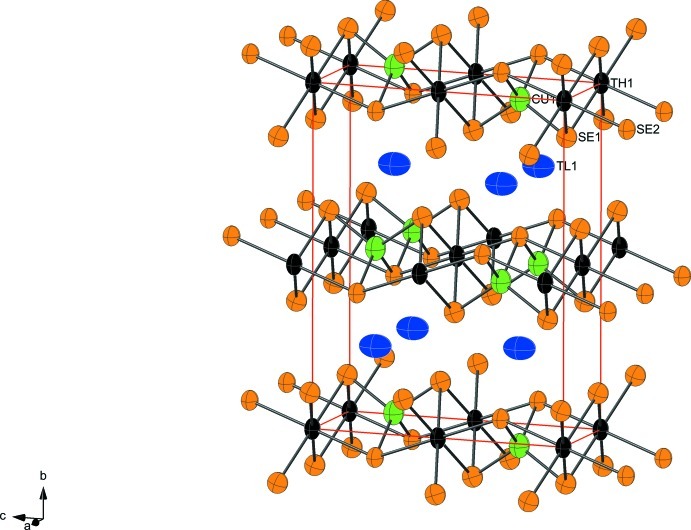
Structure of TlCuThSe3 viewed approximately down the a-axis. The 95% probability displacement ellipsoids are depicted with the unit cell outlined in red. Color key: black – Th, green – Cu, blue – Tl, orange – Se.

**Fig. 2.**
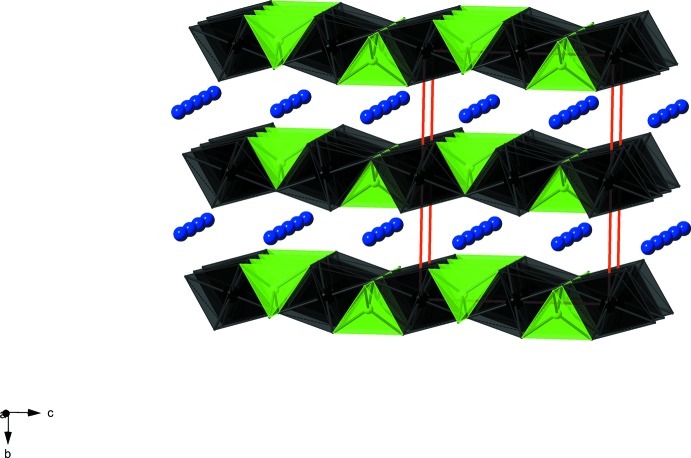
Polyhedral view of TlCuThSe3 showing sheets of edge-sharing ThSe6 octahedra (black) and CuSe4 tetrahedra (green) separated by voids filled with Tl (blue). The unit cell is outlined in red.

## supplementary crystallographic information

### Comment

Thallium(I) copper(I) thorium(IV) triselenide, TlCuThSe_3_, crystallizes in the KCuZrS_3_ structure type. The structure (Figs. 1, 2) is layered and consists of sheets of edge-sharing ThSe_6_ octahedra and CuSe_4_ tetrahedra stacked parallel to the (010) face separated by layers filled with chains of Tl running parallel to [100]. Each Tl is coordinated by a trigonal prism of Se atoms. Because there are no Se—Se bonds in the structure, oxidation states can be assigned as Tl^+^, Cu^+^, Th^4+^, and Se^2-^.

The compound TlCuThSe_3_ is of the type *AMM*'*Q*_3_, where *A* is an alkali metal or thallium, *M* is a coinage metal, *M*' is a tetravalent group IV metal or an actinide, and *Q* is a chalcogen. Including the title compound, 39 such compounds are known (Pell & Ibers, 1996; Klepp & Gurtner, 1996; Pell *et al.*, 1997; Yao *et al.*, 2008; Wells *et al.*, 2009; Bugaris & Ibers, 2009; Sutorik *et al.*, 1996; Huang *et al.*, 2001; Cody & Ibers, 1995; Mansuetto *et al.*, 1993, 1992; Klepp & Sturmayr, 1997, 1998; Seldy *et al.*, 2005; Narducci & Ibers, 2000). In all cases, crystallographic data have been collected on single crystals. Most often, the *A* site contains an alkali metal and only 6 Tl analogues are known (Pell & Ibers, 1996; Klepp & Gurtner, 1996). The *M* site contains Cu in 28 analogues, Ag in 7 analogues (Pell *et al.*, 1997; Yao *et al.*, 2008; Wells *et al.*, 2009), and Au in 4 analogues (Bugaris & Ibers, 2009). The tetravalent metal is most often U with 14 analogues (Yao *et al.*, 2008; Sutorik *et al.*, 1996; Bugaris & Ibers, 2009; Huang *et al.*, 2001; Cody & Ibers, 1995), followed by Zr with 9 analogues (Mansuetto *et al.*, 1992, 1993; Huang *et al.*, 2001; Pell *et al.*, 1997), Hf with 5 analogues (Klepp & Sturmayr, 1997, 1998; Pell *et al.*, 1997), Np with 5 analogues (Wells *et al.*, 2009), Th with 4 analogues (Seldy *et al.*, 2005; Narducci & Ibers, 2000), and Ti with 2 analogues (Mansuetto *et al.*, 1993; Pell & Ibers, 1996). This is the first compound of the type *AMM*'*Q*_3_ to contain both Tl and Th.

The compounds fall into three structure types. All the Na analogues, except for NaCuZrS_3_, are of the NaCuTiS_3_ type (space group *Pnma*) (Mansuetto *et al.*, 1993; Klepp & Sturmayr, 1997); the compounds TlCuTiTe_3_ and RbAgHfTe_3_ are of the TlCuTiTe_3_ type (space group *P*2_1_/*m*) (Pell & Ibers, 1996; Pell *et al.*, 1997); and the remaining compounds are of the KCuZrS_3_ type (space group *Cmcm*).

Interatomic distances in TlCuThSe_3_ are listed in Table 1 and are nearly identical to those in the analogues *A*CuThSe_3_ (*A* = K, Cs) (Narducci & Ibers, 2000). The TlCuThSe_3_ Th—Se distances of 2.8844 (4) and 2.9057 (5) Å match those in KCuThSe_3_ (2.893 (1) and 2.900 (1) Å) and CsCuThSe_3_ (2.878 (1) and 2.906 (1) Å). The Cu—Se distances of 2.4617 (11) and 2.5517 (11) Å also match those in KCuThSe_3_ (2.459 (2) and 2.545 (2) Å) and CsCuThSe_3_ (2.464 (2) and 2.556 (2) Å).

### Experimental

Cu (Aldrich, 99.5%), Tl_2_Se (Aldrich, 99.999%), and Se (Cerac, 99.999%) were used as received. Th chunks were powdered according to a literature procedure (Witt *et al.*, 1956). A fused-silica tube was loaded with Th (30 mg, 0.129 mmol), Cu (7.0 mg, 0.110 mmol), Tl_2_Se (36.6 mg, 0.075 mmol), and Se (20.4 mg, 0.258 mmol), evacuated to near 10 ^-4^ Torr, flame sealed, and placed in a computer-controlled furnace. It was heated to 597 K in 3 h, kept at 597 K for 24 h, heated to 1073 K in 24 h, kept at 1073 K for 96 h, cooled to 597 K in 96 h, cooled to 547 in 24 h, and then rapidly cooled to 298 K in 3 h. The reaction produced orange-red plates of TlCuThSe_3_. The elemental composition of the crystals was determined to be Tl/Cu/Th/Se in an approximate ratio of 1/1/1/3 on an EDX-equipped Hitachi S-3400 SEM.

### Refinement

The structure was standardized by means of the program *STRUCTURE TIDY* (Gelato & Parthé, 1987). The highest peak (2.0 (3) e Å^-3^) is 0.98 Å from atom Tl1 and the deepest hole (-1.3 (3) e Å^-3^) is 1.96 Å from atom Se1.

### Crystal data

**Table:**

TlCuThSe_3_	*F*(000) = 1208
*M**_r_* = 736.83	*D*_x_ = 7.611 Mg m^−^^3^
Orthorhombic, *C**m**c**m*	Mo *K*α radiation, λ = 0.71073 Å
Hall symbol: -C 2c 2	Cell parameters from 1794 reflections
*a* = 4.1678 (2) Å	θ = 2.9–28.2°
*b* = 14.2227 (7) Å	µ = 68.19 mm^−^^1^
*c* = 10.8476 (5) Å	*T* = 100 K
*V* = 643.02 (5) Å^3^	Rectangular plate, orange
*Z* = 4	0.10 × 0.07 × 0.02 mm

### Data collection

**Table:**

Bruker APEXII CCD diffractometer	474 independent reflections
Radiation source: fine-focus sealed tube	451 reflections with *I* > 2σ(*I*)
Graphite monochromator	*R*_int_ = 0.032
φ and ω scans	θ_max_ = 28.5°, θ_min_ = 2.9°
Absorption correction: numerical (*SADABS*; Sheldrick, 2008*b*)	*h* = −5→5
*T*_min_ = 0.101, *T*_max_ = 0.489	*k* = −18→18
7476 measured reflections	*l* = −14→13

### Refinement

**Table:**

Refinement on *F*^2^	Primary atom site location: structure-invariant direct methods
Least-squares matrix: full	Secondary atom site location: difference Fourier map
*R*[*F*^2^ > 2σ(*F*^2^)] = 0.021	[1.00000 + 0.00000exp(0.00(sinθ/λ)^2^)]/ [σ^2^(*F*_o_^2^) + 0.0000 + 0.0000**P* + (0.0193*P*)^2^ + 0.0000sinθ/λ] where *P* = 1.00000*F*_o_^2^ + 0.00000*F*_c_^2^
*wR*(*F*^2^) = 0.046	(Δ/σ)_max_ < 0.001
*S* = 1.59	Δρ_max_ = 2.01 e Å^−^^3^
474 reflections	Δρ_min_ = −1.34 e Å^−^^3^
24 parameters	Extinction correction: *SHELXL97* (Sheldrick, 2008*a*), Fc^*^=kFc[1+0.001xFc^2^λ^3^/sin(2θ)]^-1/4^
0 restraints	Extinction coefficient: 0.00066 (7)

### Special details

**Table:**

Geometry. All e.s.d.'s (except the e.s.d. in the dihedral angle between two l.s. planes) are estimated using the full covariance matrix. The cell e.s.d.'s are taken into account individually in the estimation of e.s.d.'s in distances, angles and torsion angles; correlations between e.s.d.'s in cell parameters are only used when they are defined by crystal symmetry. An approximate (isotropic) treatment of cell e.s.d.'s is used for estimating e.s.d.'s involving l.s. planes.
Refinement. Refinement of *F*^2^ against ALL reflections. The weighted *R*-factor *wR* and goodness of fit *S* are based on *F*^2^, conventional *R*-factors *R* are based on *F*, with *F* set to zero for negative *F*^2^. The threshold expression of *F*^2^ > σ(*F*^2^) is used only for calculating *R*-factors(gt) *etc*. and is not relevant to the choice of reflections for refinement. *R*-factors based on *F*^2^ are statistically about twice as large as those based on *F*, and *R*- factors based on ALL data will be even larger.

### Fractional atomic coordinates and isotropic or equivalent isotropic displacement parameters (Å^2^)

**Table:**

	*x*	*y*	*z*	*U*_iso_*/*U*_eq_	
Th1	0.0000	0.0000	0.0000	0.00558 (15)	
Tl1	0.0000	0.74746 (3)	0.2500	0.01247 (16)	
Se1	0.0000	0.36628 (5)	0.06410 (7)	0.0067 (2)	
Se2	0.0000	0.06909 (8)	0.2500	0.0059 (2)	
Cu1	0.0000	0.46554 (10)	0.2500	0.0085 (3)	

### Atomic displacement parameters (Å^2^)

**Table:**

	*U*^11^	*U*^22^	*U*^33^	*U*^12^	*U*^13^	*U*^23^
Th1	0.0050 (2)	0.0078 (2)	0.0040 (2)	0.000	0.000	−0.00012 (14)
Tl1	0.0101 (3)	0.0092 (3)	0.0181 (3)	0.000	0.000	0.000
Se1	0.0062 (4)	0.0071 (4)	0.0067 (4)	0.000	0.000	−0.0002 (3)
Se2	0.0069 (5)	0.0064 (5)	0.0043 (5)	0.000	0.000	0.000
Cu1	0.0100 (7)	0.0096 (7)	0.0060 (6)	0.000	0.000	0.000

### Geometric parameters (Å, º)

**Table:**

Th1—Se2^i^	2.8844 (4)	Se1—Th1^ix^	2.9057 (5)
Th1—Se2	2.8844 (4)	Se1—Th1^viii^	2.9057 (5)
Th1—Se1^ii^	2.9057 (5)	Se1—Tl1^v^	3.3564 (6)
Th1—Se1^iii^	2.9057 (5)	Se1—Tl1^iii^	3.3564 (6)
Th1—Se1^iv^	2.9057 (5)	Se1—Tl1^xii^	3.7717 (8)
Th1—Se1^v^	2.9057 (5)	Se1—Tl1^xiv^	5.6211 (6)
Th1—Cu1^iv^	3.4550 (2)	Se1—Tl1^xv^	5.6211 (6)
Th1—Cu1^v^	3.4550 (2)	Se2—Cu1^iii^	2.5517 (11)
Th1—Cu1^ii^	3.4550 (2)	Se2—Cu1^v^	2.5517 (11)
Th1—Cu1^iii^	3.4550 (2)	Se2—Th1^xvi^	2.8844 (4)
Th1—Th1^vi^	4.1678 (2)	Se2—Tl1^v^	3.2831 (9)
Th1—Th1^vii^	4.1678 (2)	Se2—Tl1^iii^	3.2831 (9)
Tl1—Se2^viii^	3.2831 (9)	Se2—Tl1^xvii^	4.5744 (12)
Tl1—Se2^ix^	3.2831 (9)	Cu1—Se1	2.4617 (11)
Tl1—Se1^x^	3.3564 (6)	Cu1—Se1^xviii^	2.4617 (11)
Tl1—Se1^viii^	3.3564 (6)	Cu1—Se2^ix^	2.5517 (11)
Tl1—Se1^xi^	3.3564 (6)	Cu1—Se2^viii^	2.5517 (11)
Tl1—Se1^ix^	3.3564 (6)	Cu1—Th1^xix^	3.4550 (2)
Tl1—Cu1^ix^	3.7368 (13)	Cu1—Th1^viii^	3.4550 (2)
Tl1—Cu1^viii^	3.7368 (13)	Cu1—Th1^xx^	3.4550 (2)
Tl1—Se1^xii^	3.7717 (8)	Cu1—Th1^ix^	3.4550 (2)
Tl1—Se1^xiii^	3.7717 (8)	Cu1—Tl1^iii^	3.7368 (13)
Tl1—Cu1	4.0095 (16)	Cu1—Tl1^v^	3.7368 (13)
Tl1—Tl1^vii^	4.1678 (2)		
			
Se2^i^—Th1—Se2	180.0	Th1^ix^—Se1—Tl1^v^	91.607 (8)
Se2^i^—Th1—Se1^ii^	89.89 (2)	Th1^viii^—Se1—Tl1^v^	156.72 (3)
Se2—Th1—Se1^ii^	90.11 (2)	Cu1—Se1—Tl1^iii^	78.26 (3)
Se2^i^—Th1—Se1^iii^	90.11 (2)	Th1^ix^—Se1—Tl1^iii^	156.72 (3)
Se2—Th1—Se1^iii^	89.89 (2)	Th1^viii^—Se1—Tl1^iii^	91.607 (8)
Se1^ii^—Th1—Se1^iii^	180.00 (4)	Tl1^v^—Se1—Tl1^iii^	76.761 (17)
Se2^i^—Th1—Se1^iv^	89.89 (2)	Cu1—Se1—Tl1^xii^	170.40 (4)
Se2—Th1—Se1^iv^	90.11 (2)	Th1^ix^—Se1—Tl1^xii^	93.702 (18)
Se1^ii^—Th1—Se1^iv^	91.64 (2)	Th1^viii^—Se1—Tl1^xii^	93.702 (18)
Se1^iii^—Th1—Se1^iv^	88.36 (2)	Tl1^v^—Se1—Tl1^xii^	109.076 (17)
Se2^i^—Th1—Se1^v^	90.11 (2)	Tl1^iii^—Se1—Tl1^xii^	109.076 (17)
Se2—Th1—Se1^v^	89.89 (2)	Cu1—Se1—Tl1^xiv^	131.422 (8)
Se1^ii^—Th1—Se1^v^	88.36 (2)	Th1^ix^—Se1—Tl1^xiv^	125.11 (2)
Se1^iii^—Th1—Se1^v^	91.64 (2)	Th1^viii^—Se1—Tl1^xiv^	60.762 (10)
Se1^iv^—Th1—Se1^v^	180.00 (4)	Tl1^v^—Se1—Tl1^xiv^	132.816 (19)
Se2^viii^—Tl1—Se2^ix^	78.80 (3)	Tl1^iii^—Se1—Tl1^xiv^	76.051 (8)
Se2^viii^—Tl1—Se1^x^	141.551 (14)	Tl1^xii^—Se1—Tl1^xiv^	47.856 (6)
Se2^ix^—Tl1—Se1^x^	89.713 (15)	Cu1—Se1—Tl1^xv^	131.422 (9)
Se2^viii^—Tl1—Se1^viii^	89.713 (15)	Th1^ix^—Se1—Tl1^xv^	60.763 (10)
Se2^ix^—Tl1—Se1^viii^	141.551 (14)	Th1^viii^—Se1—Tl1^xv^	125.11 (2)
Se1^x^—Tl1—Se1^viii^	119.54 (3)	Tl1^v^—Se1—Tl1^xv^	76.051 (8)
Se2^viii^—Tl1—Se1^xi^	89.713 (15)	Tl1^iii^—Se1—Tl1^xv^	132.816 (19)
Se2^ix^—Tl1—Se1^xi^	141.551 (14)	Tl1^xii^—Se1—Tl1^xv^	47.856 (6)
Se1^x^—Tl1—Se1^xi^	76.760 (17)	Tl1^xiv^—Se1—Tl1^xv^	95.712 (12)
Se1^viii^—Tl1—Se1^xi^	73.86 (2)	Cu1^iii^—Se2—Cu1^v^	109.50 (7)
Se2^viii^—Tl1—Se1^ix^	141.551 (14)	Cu1^iii^—Se2—Th1	78.662 (19)
Se2^ix^—Tl1—Se1^ix^	89.713 (15)	Cu1^v^—Se2—Th1	78.662 (19)
Se1^x^—Tl1—Se1^ix^	73.86 (2)	Cu1^iii^—Se2—Th1^xvi^	78.662 (19)
Se1^viii^—Tl1—Se1^ix^	76.760 (17)	Cu1^v^—Se2—Th1^xvi^	78.662 (19)
Se1^xi^—Tl1—Se1^ix^	119.54 (3)	Th1—Se2—Th1^xvi^	140.17 (4)
Se2^viii^—Tl1—Se1^xii^	70.645 (11)	Cu1^iii^—Se2—Tl1^v^	164.65 (4)
Se2^ix^—Tl1—Se1^xii^	70.645 (11)	Cu1^v^—Se2—Tl1^v^	85.85 (3)
Se1^x^—Tl1—Se1^xii^	139.351 (12)	Th1—Se2—Tl1^v^	105.262 (13)
Se1^viii^—Tl1—Se1^xii^	70.924 (17)	Th1^xvi^—Se2—Tl1^v^	105.262 (13)
Se1^xi^—Tl1—Se1^xii^	139.351 (11)	Cu1^iii^—Se2—Tl1^iii^	85.85 (3)
Se1^ix^—Tl1—Se1^xii^	70.924 (17)	Cu1^v^—Se2—Tl1^iii^	164.65 (4)
Cu1^ix^—Tl1—Se1^xii^	110.855 (11)	Th1—Se2—Tl1^iii^	105.262 (13)
Cu1^viii^—Tl1—Se1^xii^	110.855 (11)	Th1^xvi^—Se2—Tl1^iii^	105.262 (13)
Se2^viii^—Tl1—Se1^xiii^	70.645 (11)	Tl1^v^—Se2—Tl1^iii^	78.80 (3)
Se2^ix^—Tl1—Se1^xiii^	70.645 (11)	Cu1^iii^—Se2—Tl1^xvii^	54.75 (3)
Se1^x^—Tl1—Se1^xiii^	70.924 (17)	Cu1^v^—Se2—Tl1^xvii^	54.75 (3)
Se1^viii^—Tl1—Se1^xiii^	139.351 (11)	Th1—Se2—Tl1^xvii^	70.08 (2)
Se1^xi^—Tl1—Se1^xiii^	70.924 (17)	Th1^xvi^—Se2—Tl1^xvii^	70.08 (2)
Se1^ix^—Tl1—Se1^xiii^	139.351 (11)	Tl1^v^—Se2—Tl1^xvii^	140.600 (14)
Cu1^ix^—Tl1—Se1^xiii^	110.855 (11)	Tl1^iii^—Se2—Tl1^xvii^	140.600 (14)
Cu1^viii^—Tl1—Se1^xiii^	110.855 (11)	Se1—Cu1—Se1^xviii^	110.01 (7)
Se1^xii^—Tl1—Se1^xiii^	129.21 (3)	Se1—Cu1—Se2^ix^	109.328 (14)
Cu1—Se1—Th1^ix^	79.67 (3)	Se1^xviii^—Cu1—Se2^ix^	109.328 (14)
Cu1—Se1—Th1^viii^	79.67 (3)	Se1—Cu1—Se2^viii^	109.328 (14)
Th1^ix^—Se1—Th1^viii^	91.64 (2)	Se1^xviii^—Cu1—Se2^viii^	109.328 (14)
Cu1—Se1—Tl1^v^	78.26 (3)	Se2^ix^—Cu1—Se2^viii^	109.51 (7)

Symmetry codes: (i) −*x*, −*y*, −*z*; (ii) −*x*+1/2, −*y*+1/2, −*z*; (iii) *x*−1/2, *y*−1/2, *z*; (iv) −*x*−1/2, −*y*+1/2, −*z*; (v) *x*+1/2, *y*−1/2, *z*; (vi) *x*−1, *y*, *z*; (vii) *x*+1, *y*, *z*; (viii) *x*−1/2, *y*+1/2, *z*; (ix) *x*+1/2, *y*+1/2, *z*; (x) *x*+1/2, *y*+1/2, −*z*+1/2; (xi) *x*−1/2, *y*+1/2, −*z*+1/2; (xii) −*x*, −*y*+1, −*z*; (xiii) −*x*, −*y*+1, *z*+1/2; (xiv) −*x*−1, −*y*+1, −*z*; (xv) −*x*+1, −*y*+1, −*z*; (xvi) −*x*, −*y*, *z*+1/2; (xvii) *x*, *y*−1, *z*; (xviii) *x*, *y*, −*z*+1/2; (xix) −*x*+1/2, −*y*+1/2, *z*+1/2; (xx) −*x*−1/2, −*y*+1/2, *z*+1/2.
